# 4th booster-dose SARS-CoV-2 heterologous and homologous vaccination in rheumatological patients

**DOI:** 10.3389/fimmu.2024.1427501

**Published:** 2024-07-26

**Authors:** Maria Jose Gallardo-Nelson, Marcos Cruces, Yolanda M. Gómez, Constanza Fuenzalida, Javiera Silva, Laura Aravena-Traipi, Eduardo Nuñez, Aracelly Gaete-Angel, Elizabeth Rivas-Yañez, Alexis M. Kalergis, Ricardo Soto-Rifo, Fernando Valiente-Echeverria

**Affiliations:** ^1^ Departamento de Medicina, Facultad de Medicina, Universidad de Atacama, Copiapó, Chile; ^2^ Policlínico Reumatología e Inmunología, Hospital Regional de Copiapó, Copiapó, Chile; ^3^ Departamento de Estadística, Facultad de Ciencias, Universidad del Bío-Bío, Concepción, Chile; ^4^ Laboratory of Molecular and Cellular Virology, Virology Program, Institute of Biomedical Sciences, Faculty of Medicine, Universidad de Chile, Santiago, Chile; ^5^ Millennium Institute on Immunology and Immunotherapy, Santiago, Chile; ^6^ Facultad de Ciencias Biológicas, Pontificia Universidad Católica de Chile, Santiago, Chile; ^7^ Departamento de Endocrinología, Facultad de Medicina, Escuela de Medicina, Pontificia Universidad Católica de Chile, Santiago, Chile

**Keywords:** SARS-CoV-2, COVID-19 vaccines, autoimmune rheumatic diseases, humoral IgG, immunosuppressive therapies

## Abstract

**Objective:**

to evaluate the immune response to the SARS-CoV-2 vaccines in adults with immune-mediated rheumatic diseases (IMRDs) in comparison to healthy individuals, observed 1-20 weeks following the fourth vaccine dose. Additionally, to evaluate the impact of immunosuppressive therapies, vaccination schedules, the time interval between vaccination and sample collection on the vaccine’s immune response.

**Methods:**

We designed a longitudinal observational study conducted at the rheumatology department of Hospital de Copiapó. Neutralizing antibodies (Nabs) titers against the Wuhan and Omicron variant were analyzed between 1-20 weeks after administration of the fourth dose of the SARS-CoV-2 vaccine to 341 participants (218 IMRD patients and 123 healthy controls). 218 IMRD patients with rheumatoid arthritis (RA), psoriatic arthritis (PsA), ankylosing spondylitis (AS), systemic lupus erythematosus (SLE), systemic vasculitis (VS) and systemic scleroderma (SS) were analyzed.

**Results:**

Performing a comparison between the variants, Wuhan vs Omicron, we noticed that there were significant differences (p<0.05) in the level of the ID_50_, both for healthy controls and for patients with IMRDs. The humoral response of patients with IMRDs is significantly lower compared to healthy controls for the Omicron variant of SARS-CoV-2 (p = 0.0015). The humoral response of patients with IMRDs decreases significantly when the time interval between vaccination and sample collection is greater than 35 days. This difference was observed in the response, both for the Wuhan variant and for the Omicron variant.

**Conclusion:**

The IMRDs patients, the humoral response variation in the SARS-CoV-2 vaccine depends on doses and type of vaccine administered, the humoral response times and the treatment that these patients are receiving.

## Introduction

The worldwide pandemic of the severe acute respiratory syndrome coronavirus 2 (SARS-CoV-2) has resulted in over 6 million fatalities (1). In Chile, until December 25, 2022, there were 5,366,630 cases of COVID-19 (2). It was critically important to prevent the spread of the COVID-19 pandemic. An increasing variety of SARS-CoV-2 vaccines are being utilized globally, encompassing mRNA-based, adenoviral vector, protein subunit, and inactivated virus vaccines (3). The immunogenicity of the SARS-CoV-2 vaccine can be quantified through the measurement of humoral IgG to the spike protein or cellular T-cell reactivity through interferon (IFN)-γ response towards SARS-CoV-2 peptides. The antibody responses are commonly recorded as ‘seroconversion’ (freshly positive anti-spike protein IgG), or via post-immunization antibody levels (4). In Chile, the effectiveness of booster shots against symptomatic COVID-19 was measured, with 56-80% for CoronaVac (Sinovac Biotech), 56-90% for BNT162b2 (Pfizer-BioNTech) and 56-93% for ChAdOx1-S (AstraZeneca-Oxford). The effectiveness against hospitalization 14 days after the booster shot was 84-88%, 84-87% and 84-96% respectively (5).

The Omicron variant (B.1.1.529 lineage) was the most frequent variant in the Chilean population during January 2022, representing 71.8% (6). Patients with immune-mediated rheumatic diseases (IMRDs) are at increased risk of serious infections because of the subyacent dysregulation of their immune system and the common use of immunosuppressors and antirheumatic biological therapies (7). Decreased reactions to traditional vaccines are linked with advanced age, high-dose steroid use, elevated inflammatory activity index, and immunosuppressive treatments (8). Findings indicate that in patients with rheumatic diseases, the responses to the SARS-CoV-2 vaccine are compromised by rituximab, glucocorticoids (GC), methotrexate, abatacept, mycophenolate mofetil, and JAK inhibitors (9–12). In Chile, diverse immunocompromising conditions markedly reduce the humoral response to the CoronaVac vaccine (13). The present study aimed to evaluate the immune response induced by 4th dose of different SARS-CoV-2 vaccines, measuring levels of neutralizing antibodies (NAbs) in adult patients with IMRDs and comparing them with healthy controls.

## Methodology

This longitudinal observational study was carried out in the rheumatology department of the Regional Hospital of Copiapó (Atacama, Chile) between March and July 2022. The primary objective was to assess the immunogenicity of SARS-CoV-2 vaccines in adult patients with IMRD compared to healthy controls, measured between 1 and 20 weeks after the fourth vaccine dose. Additionally, other secondary objectives were analyzed, such as the immunogenic effect of the original Wuhan strain and its Omicron variant in healthy controls and patients, the SARS-CoV-2 vaccination schedules implemented in the country, the kinetics of the humoral response to SARS-CoV-2 vaccination, and the effect of immunosuppressive pharmacological treatments and specific pathologies (RA, PsA, AS, VS, and SS) on the immunogenicity of the vaccine in patients with IMRD.

### Study population

A non-probabilistic convenience sampling was carried out in the population with IMRDs population over 18 years from the rheumatology outpatient clinic at Hospital de Copiapó. Patients with 4^th^ dose SARS-CoV-2 vaccine were included. Selected diseases were rheumatoid arthritis (RA), psoriatic arthritis (PsA), ankylosing spondylitis (AS), systemic lupus erythematosus (SLE), systemic vasculitis (VS) and systemic scleroderma (SS). Patients continued with their usual medication during the vaccination period, in certain cases, patients using rituximab were instructed to delay treatment after vaccination at the doctor’s discretion.

**Figure 1:**
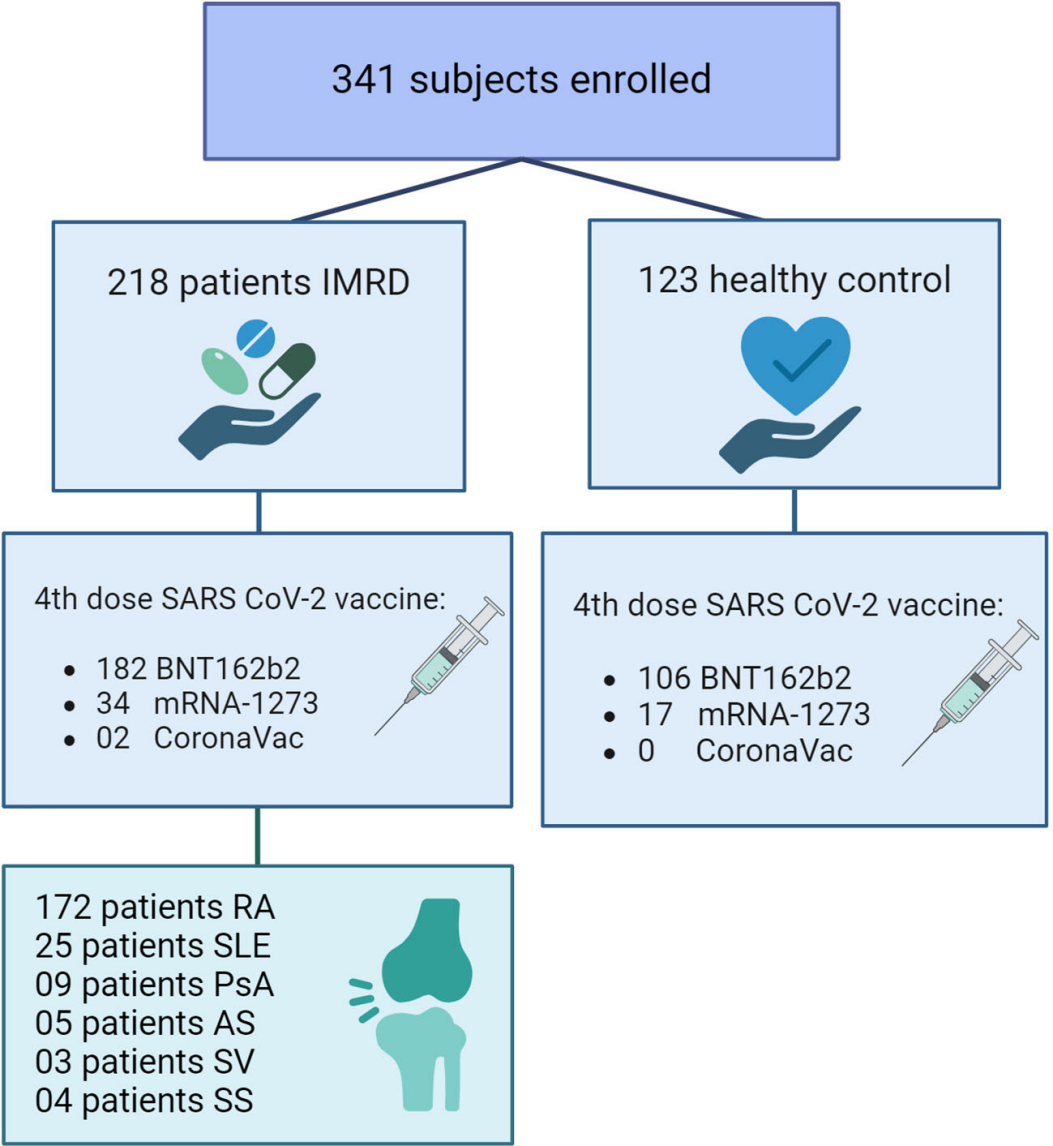
Flow diagram of the population participating in the study. IMRDs, immune-mediated rheumatic diseases. RA, Rheumatoid arthritis; SLE, Systemic lupus erythematosus; PsA, Psoriatic arthritis; AS, Ankylosing spondylitis; SV, Systemic Vasculitis; SS, Systemic scleroderma.

Participants without immunosuppression vaccinated with 4^th^ dose against SARS-CoV2 during the same timeframe, were chosen for the control group. The control group consisted predominantly of health care professionals, providing a representative sample of the broader population (**Figure 1**).

General exclusion criteria were previous serologically or PCR-confirmed COVID-19, and in the control group, a history of IMRDs and immunosuppressive treatment.

The subsequent factors were documented: age, sex, type of rheumatic disease, a brand of vaccine administered, time since diagnosis, current treatment with synthetic disease-modifying antirheumatic drugs (DMARDs), biological disease-modifying antirheumatic drugs (bDMARDs) and or JAK inhibitors, glucocorticoid dose (prednisone equivalent), time since last infusion in rituximab or cyclophosphamide treated patients, safety of vaccination and comorbidities.

### Vaccination procedure

All patients and controls received a 4^th^ dose of the SARS-CoV-2 vaccine with BNT162b2, CoronaVac or mRNA-1273 vaccines (6). Samples for measured immunogenicity were taken 1–20 weeks after vaccination.

### Determination of Nabs against SARS-CoV-2

We conducted an analysis of the neutralizing antibody levels against SARSCoV-2 D614G (B.1 lineage) and Omicron (BA.1 lineage) by utilizing an HIV-1–SΔ19 pseudotype (SARS-CoV-HIV-luciferase). This method facilitates large-scale detection and quantification of the existence of neutralizing antibodies (Nabs) (14). Serum samples that were inactivated underwent a 3-fold serial dilution, ranging from 1:5 to 1:10935, in DMEM that was supplemented with 10% FBS. These samples were then incubated with 3 ng of p24 HIV-1-based SARS-CoV-2 variant pseudotyped virus hailing from either the Wuhan (B.1 lineage) or Omicron (BA.1 lineage)), for a period of 1 hour at a temperature of 37°C. Following this, 1 × 104 HEK-ACE2 cells were introduced into each well. As a negative control, HEK293T cells were incubated with the pseudotyped virus. After 48 hours, cells were broken down, and the firefly luciferase activity was assessed using the Luciferase Assay Reagent (Promega) in a Glomax 96 Microplate luminometer (Promega). The average relative luminescence units (RLUs) of HEK293T cells infected with the equivalent pseudovirus were assumed as 100% neutralization, while RLUs recorded at each sample’s highest dilution were set as 0% neutralization. Consequently, the neutralization percentage of each of the eight dilutions of a sample was determined as the complement of the division between the related RLUs and the RLUs achieved at the higher dilution after subtracting the background (HEK293T + pseudovirus). This calculation was performed individually for each technical replicate and for each spike variant. The Relative pseudotyped Virus Neutralization Titer 50 (ID_50_) is a term used to describe the sample dilution required to decrease firefly luciferase activity by 50% when compared to both positive and negative controls. The negative control is the HEK293T without the pseudovirus and the positive control is the highest sample dilution. The calculation of the ID_50_ was performed using GraphPad Prism v9.1.2, a software-based in La Jolla, California, USA, by applying a four-parameter non-linear regression model with a variable slope. This model curbs the highest values at 100 and the lowest at 0. If a sample displayed a pVNT_50_ value that was lower than the first dilution (1:10), it was noted as 10 (15, 16). **Figure 1** of the **Supplementary Material** is a graphical explanation of the method to explain neutralizing antibodies determination.

### Statistical analysis

Variables in categories were presented in percentage form, while continuous variables were depicted as median and Interquartile Range (IQR) values. The Mann-Whitney U test was employed to evaluate the distinctions between groupings. Comparison of categorical variables was achieved using contingency tables, and p values were determined with either χ2 or Fisher’s exact tests, as the situation required. P values of ≤0.05 were considered statistically significant. All the given p values are two-tailed. These data were entered and administered using the REDCap database tool housed at the Faculty of Medicine, University of Chile (17), statistical analyses were performed using the R software version 4.3.1 (18) and GraphPad Prism version 8.4.3.

### Ethics

The study was carried out following the principles of the Declaration of Helsinki and was approved by the ethics committee of the Faculty of Medicine of the University of Chile (Project N° 161-2021). All participants signed informed consent before inclusion in the study.

## Results

### Demographic characteristics of the study population

A total of 341 participants were enrolled, of which 218 were patients with IMRDs and 123 healthy controls. The demographic characteristics of the group finally analyzed are given in **Table 1**.

**Table 1:** Demographic characteristics of patients with IMRDs and controls.

	n	Age median (IQR)	Female (%)	Disease Duration median (IQR)	Time window median (IQR)
**Controls**	123	39 (24-51)	63.4	NA	38 (24-57)
**IMRDs diagnosis**	218	58 (48.25-64)	84.8	6 (4-10)	31(22-51)
**Rheumatoid arthritis (RA)**	172	58 (51-64)	89.5	7 (5-11)	37.5(24.75-59)
**Systemic Lupus Erythematosus (SLE)**	25	47 (34-56)	88	6 (3-9)	35(20-44)
**Psoriatic Arthritis (PsA)**	9	50 (35-56)	33.33	3 (2-5)	32(24-72)
**Ankylosing Spondylitis (AS)**	5	37 (32-40)	60	3 (1-3)	42(26-51)
**Systemic Vasculitis (VS)**	3	59 (55-67)	33.33	6 (4-7.5)	39(27.5-55.5)
**Systemic Scleroderma (SS)**	4	59 (55-67)	50	5.5 (1.75-9)	43.5(33.5-59.75)

Immune-mediated rheumatic diseases (IMRDs).

The median age of healthy controls was 39 years, while for IMRDs patients was 58 years. Regarding gender distribution, 63.4% of healthy controls were women, while IMRDs patients were 84.8%. RA was the most common disease among participants (n=172), followed by SLE (n=25). The median duration of the rheumatic disease in patients was 6 years. Samples are measured 1-20 weeks after vaccination, the median of the time window is 35 days.

### Immunogenicity of vaccination against SARS-CoV-2

The fourth dose of SARS-CoV-2 vaccination was mostly administered with BNT162b2, both for healthy controls and IMRDs patients. The NAbs titer against SARS-CoV-2 was determined for the original Wuhan strain and Omicron variant, using the methodology for the detection of antibodies with neutralizing capacity described in the work by Beltrán-Pavez et al. (2021) (14). The presence of NAbs against protein S of the Wuhan strain was 100% for healthy controls and 99.5% for rheumatic patients. For the Omicron variant, it was 100% for healthy controls and 99% for rheumatic patients.

**Figure 2:**
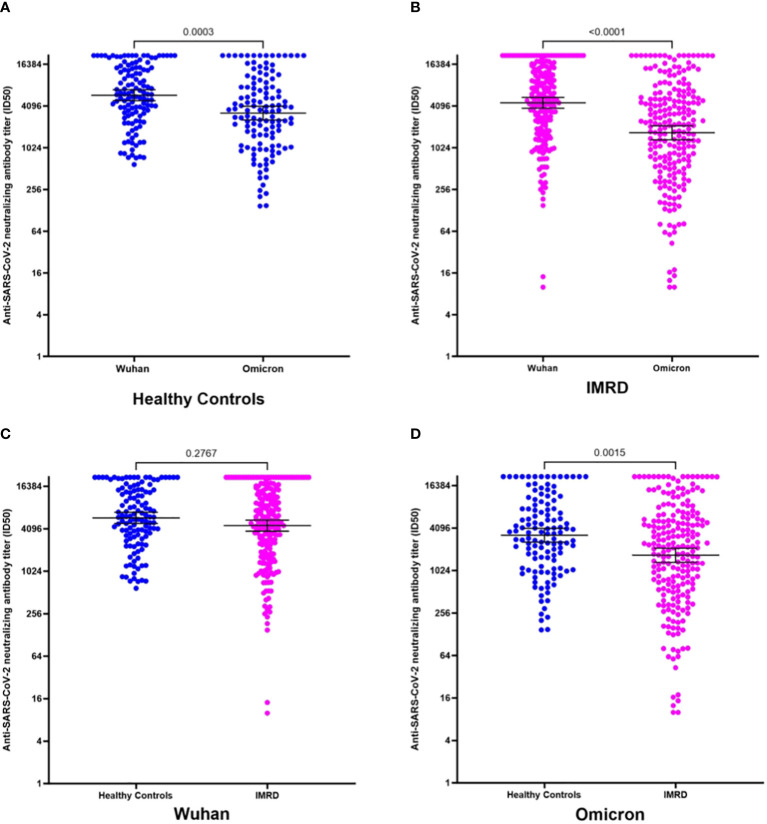
NAbs titer against SARS-CoV-2 for the Wuhan strain and Omicron variant, according to the time interval between vaccination and sample collection. **(A)** ID_50_ neutralizing antibodies of healthy controls according to the post-vaccination time, for the Wuhan strain. **(B)** ID_50_ of NAbs from IMDRs patients according to time post-vaccination for the Wuhan strain. **(C)** ID_50_ of NAbs from healthy controls according to time post-vaccination for the Omicron variant. **(D)** ID_50_ of NAbs from IMDRs patients according to time post vaccination for the Omicron variant.

**Figure 3:**
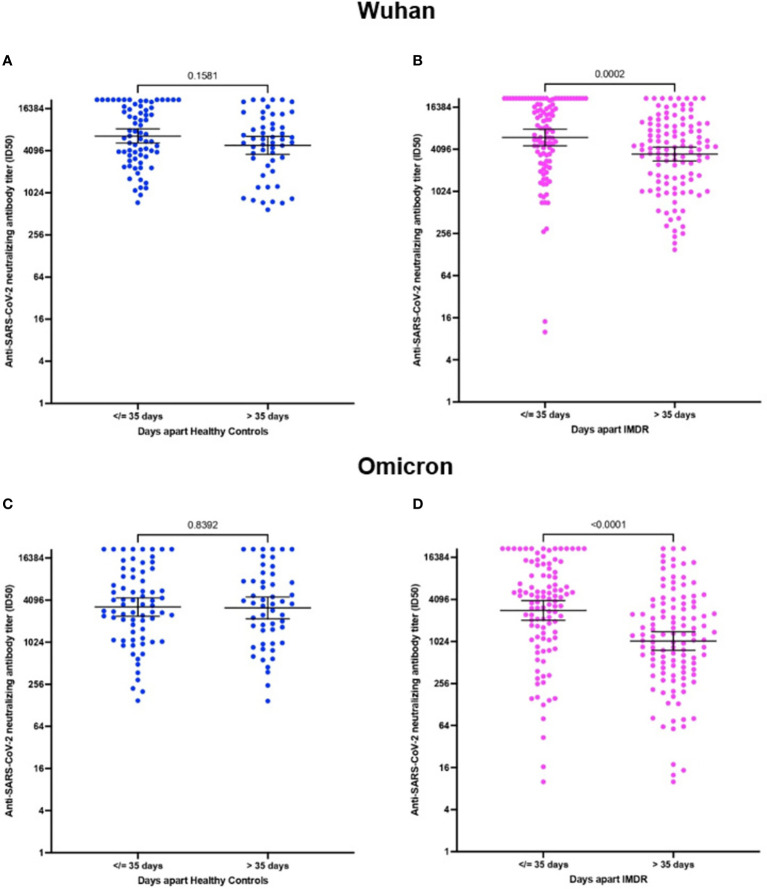
NAbs titer against SARS-CoV-2 for the original Wuhan strain and the omicron variant, in the serum of rheumatic patients and healthy controls after the fourth dose of vaccination against SARS-CoV-2. **(A)** ID_50_ for the original Wuhan strain and the omicron variant in the serum of healthy controls. **(B)** ID_50_ for the original Wuhan strain and the omicron variant in the serum of IMRDs patients. **(C)** ID_50_ of NAbs in healthy controls and IMRDs patients for the original Wuhan strain. **(D)** ID_50_ of NAbs in healthy controls and IMRDs patients for the Omicron variant.

The NAbs titer in patients and healthy controls presented a higher titer for the original Wuhan strain than for the Omicron variant (**Figures 2A, B**). The median ID_50_ for the Wuhan strain was 6030 ± 7285.5 versus 3533 ± 6912.3 for the Omicron variant (p=0.0003) for healthy controls (**Figure 3A**). In IMRDs patients the median ID_50_ was 5298 ± 7514.3 for the Wuhan strain compared to 2144.5 ± 6354.8 for the Omicron variant (p < 0.001) (**Figure 3B**). The humoral response of IMRDs patients is significantly lower compared to healthy controls for the Omicron variant of SARS-CoV-2 (p=0.0015) (**Figure 2D**), but not in the NAbs titer for the Wuhan strain (**Figure 3C**).

The measurement of NAbs was performed between 1 and 20 weeks after vaccination of the fourth dose. This allows us to observe the humoral response in the time interval between the administration of the vaccine and the sample collection, the median of the time window is 35 days, we choose this endpoint of 35 days to ensure 50% of the samples were in each group. Besides in this case there is no significant difference (p-value>0.05), therefore we have no differences between the times since vaccination and sampling between patients and healthy controls.

The NAbs titer of patients with IMRDs decreases significantly when the time interval between vaccination and sample collection is greater than 35 days (**Figure 2**). This difference is observed in the response, both for the original strain from Wuhan (**Figure 2B**) and for the Omicron variant (**Figure 2D**). In contrast, in healthy controls, the humoral response is maintained over time, both for intervals less than or equal to 35 days and for intervals of more than 35 days (**Figures 2A–C**), in response to both strains.

### Immunogenicity according to the vaccination scheme used against SARS-CoV-2

Study participants have different vaccination schedules against SARS-CoV-2. Homologous schemes are defined as those that use the BNT162b2 and mRNA-1273 vaccines since they present the same vaccination strategy through mRNA. Heterologous schemes are defined as mixed schemes, where CoronaVac vaccines were used for the first 2 doses and then different combinations with vaccines such as BNT162b2, ChAdOx1-S or mRNA-1273. Healthy controls with homologous schemes represent 54% and with heterologous schemes 46%. In IMRDs patients the heterologous scheme was the majority with 68% of the patients and 32% with a homologous scheme.

**Figure 4:**
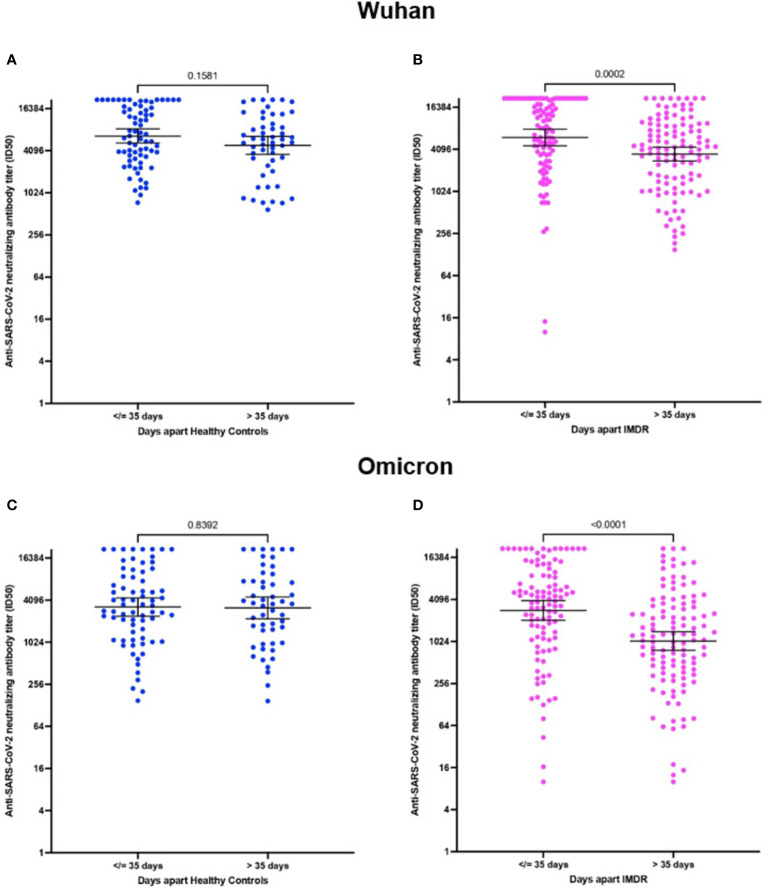
NAbs titer against SARS-CoV-2 in the serum of rheumatic patients and healthy controls according to the vaccination scheme used against SARS-CoV-2. **(A)** ID_50_ of NAbs against SARS-CoV-2 between IMDRs patients and healthy controls for the Wuhan strain. **(B)** ID_50_ of NAbs against SARS-CoV-2 according to the vaccination scheme for the Wuhan strain. **(C)** ID_50_ of NAbs against SARS-CoV-2 between IMDRs patients and healthy controls for the Omicron variant. **(D)** ID_50_ of NAbs against SARS-CoV-2 according to the vaccination schedule for the Omicron variant.

In homologous vaccination schemes, no differences in their NAbs titers against SARS-CoV-2 were observed between healthy controls and IMRDs patients (**Figures 4A–C**). In contrast, in heterologous schemes, the NAbs titer against SARS-CoV-2 was lower in IMRDs patients than in healthy controls, in response to the Omicron variant (p =0.0149) (**Figure 4C**), which is not observed in response to the Wuhan strain (**Figure 4A**).

The NAbs titer against SARS-CoV-2 in the serum of patients who followed a heterologous regimen is significantly lower than that of patients who followed a homologous regimen. Results were observed for the original Wuhan strain (p =0.0186) (**Figure 4B**) and for the Omicron variant (p = 0.0014) (**Figure 4D**). In healthy controls, the NAbs titers against SARS-CoV-2 do not show differences between the different vaccination schemes.

### Immunogenicity according to IMRDs

**Figure 5:**
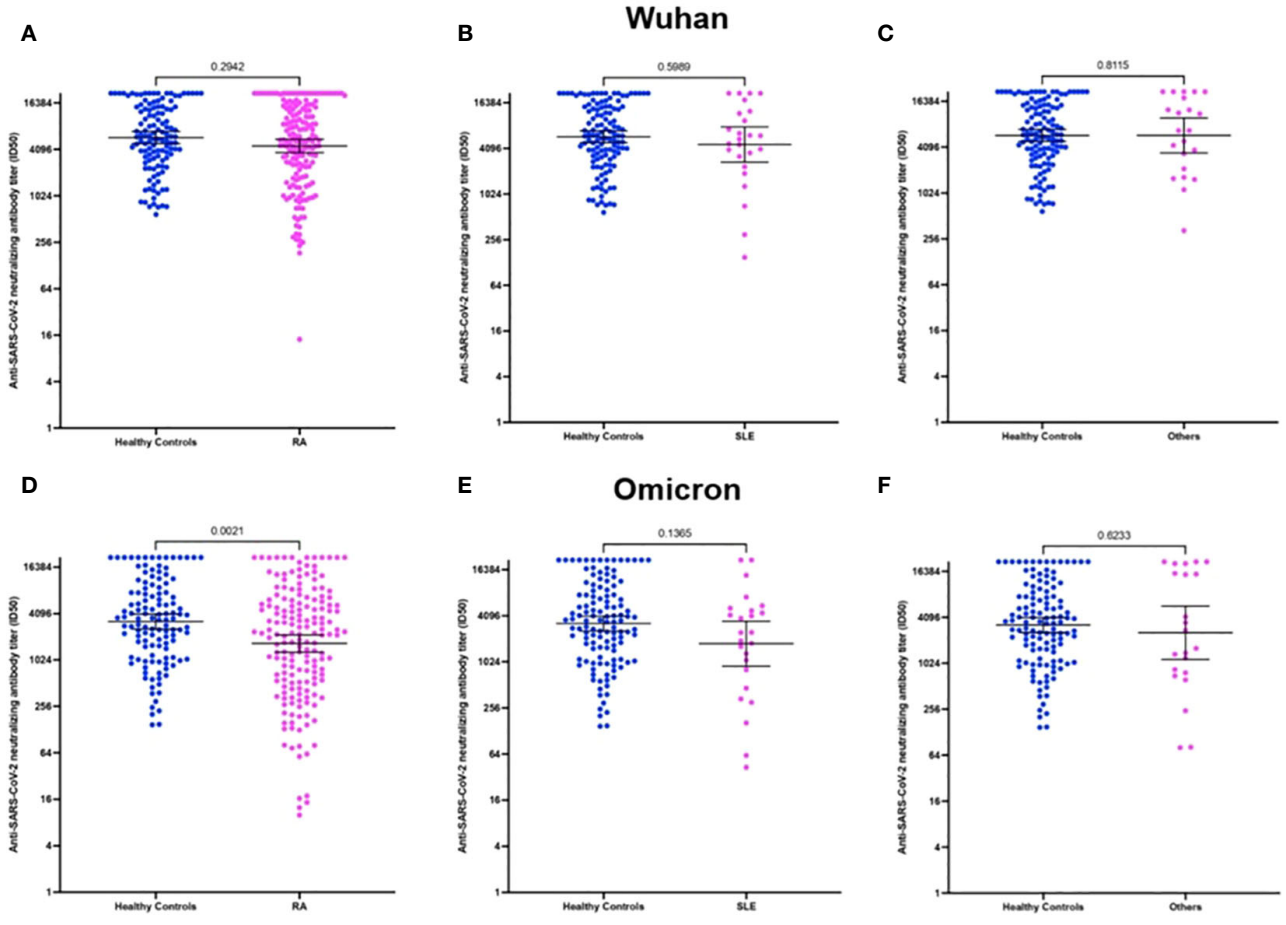
NAbs titer against SARS-CoV-2 in the serum of patients with rheumatoid arthritis (RA), Systemic lupus erythematosus (SLE) and other rheumatic diseases included in the study. **(A)** ID_50_ of neutralizing antibodies against SARS-CoV-2 from RA patients against the Wuhan strain. **(B)** ID_50_ of NAbs against SARS-CoV-2 from SLE patients against the Wuhan strain. **(C)** ID_50_ of NAbs against SARS-CoV-2 from patients with other rheumatic diseases against the Wuhan strain. **(D)** ID_50_ of NAbs against SARS-CoV-2 from RA patients against the Omicron variant. **(E)** ID_50_ of NAbs against SARS-CoV-2 from SLE patients against the Omicron variant. **(F)** ID_50_ of NAbs against SARS-CoV-2 from patients with other rheumatic diseases against the Omicron variant.

In our patients with IMRDs, the main disease represented is RA. The humoral response of RA patients against the parental Wuhan strain did not show differences compared to healthy controls (**Figure 5A**). Instead, when analyzing the response of these patients against the Omicron variant, a significant decrease in the NAbs titers was observed compared to healthy controls (**Figure 5D**). When analyzing the response to the antibody titer of SLE patients compared to healthy controls, no significant differences were observed, neither for the Wuhan strain nor for the Omicron (**Figures 5B–E**). The other IMRDs included in our study are PsA, AS, SV and SS. These diseases were analyzed together due to their small sample size, without observing significant differences in the titer of NAbs against the Wuhan strain or Omicron variant compared to healthy controls (**Figures 5C–F**).

### Effect of pharmacological treatments on the immune response

In participating IMRDs patients, 72% are treated with GC (approximately 5 mg/day) (**Table 2**). We can observe that these patients present a significant decrease in the titer of NAbs for SARS-CoV-2 in response to vaccination, both for the original Wuhan strain and for the Omicron variant.

**Table 2:** Immunogenicity of vaccination against SARS-CoV-2 according to the treatments used by IMRDs patients compared to healthy controls.

Treatments, n	Wuhan		Omicron	
	MEDIAN ± SD	P value	MEDIAN ± SD	P value
**Healthy Control, n=123**	6030.0 (± 7285,5)	–	3533.0 (± 6912.3)	–
**GC, n=156**	5395.0 (± 7450.3)	0.009***	2143.0 (± 6527.6)	0.0018***
**MTX total, n=98**	5517.0 (± 7876.4)	0.014**	2196.0 (± 6388.7)	0.03342**
**MTX monotherapy, n=15**	5455.0 (± 7249.6)	0.6123	2293.0 (± 4013.1)	0.4037
**MTX + GC, n=27**	4539.0 (± 8272.5)	0.2945	2077.0 (± 6987.9)	0.09457*
**LEF, n=83**	5568.0 (± 7242.7)	0.04312**	1701.0 (± 5918.2)	0.0006***
**LEF + GC, n=15**	5053.0 (± 6700.3)	0.6317	1129.0 (± 4756.5)	0.02434**
**HCQ, n=65**	5344.0 (± 7484.9)	0.05469*	2281.0 (± 6636.4)	0.01772**
**HCQ monotherapy, n=8**	3816.0 (± 2253.7)	0.6204	2844.5 (± 7290.9)	0.4108
**Sulfadiazine, n=22**	7656.0 (± 7509.1)	0.08678*	3440.0 (± 7390.6)	0.6631
**Mycophenolate, n=12**	3284.0 (± 6488.5)	0.9969	1350.0 (± 1703.8)	0.008435***
**Azathioprine, n=7**	4724.0 (± 8552.8)	0.7102	1918.0 (± 2321.9)	0.1372
**Anti TNF, n=35**	5155.0 (± 7625.1)	0.669	3129.0 (± 7603.9)	0.4068
**Anti TNF monotherapy, n=6**	10852.0 (± 7841.2)	0.4042	10739.5 (± 8222.8)	0.200
**Rituximab, n=11**	2811.0 (± 8253.2)	0.4653	429.7 (± 1412.6)	0.00053***
**Abatacept, n=6**	10502.0 (± 9201.7)	0.5493	553.1 (± 1433.9)	0.0084***
**Secukinumab, n=6**	11368.0 (± 9057.7)	0.144	2183.0 (± 10487.9)	0.9777

The comparison was performed based on the Mann-Whitney U test.

GC, Glucocorticoids; MTX, Methotrexate; LEF, Leflunomide; HCQ, hydroxychloroquine; TNF, Tumor necrosis factor; SD, standard deviation. *p ≤ 0.1; ** p ≤ 0.05; ***p ≤ 0.01.

Likewise, methotrexate (MTX) and leflunomide (LEF) treatment is widely used by IMRDs patients (mainly in RA patients), either as monotherapy or in combination with other treatments. The titer of NAbs against SARS-CoV-2 of patients treated with MTX or LEF in combination with other treatments significantly decreased compared to healthy controls both for the original Wuhan strain and for the Omicron variant. Treatments such as Hydroxychloroquine (in combination), mycophenolate, rituximab and abatacept also show a decrease in NAbs titer against SARS-CoV-2, but only in response to the Omicron variant (**Table 2**).

## Discussion

Our data reveal that both healthy controls and IMRDs patients have high seropositivity and high NAbs titer against the original SARS-CoV-2 Wuhan strain and the Omicron variant. This could be attributed to the extensive vaccination campaign carried out by the Chilean Ministry of Health, which contemplated four vaccination doses until the development of this study, intending to maintain high antibody titers against SARS-CoV-2 and to control the infection of the Omicron variant strain in 2022 (6, 19–21). These data are consistent with recent studies carried out in a population vaccinated against SARS-CoV-2, plus one or two booster doses, where it was observed that 98.2% of people had NAbs against the Wuhan variant, while 65.5% had NAbs against the Omicron variant (22).

The Omicron variant was the most frequent in the Chilean population during January 2022 (6). This variant presents 36 mutations in the S (spike) protein, which is the immunological target against NAbs (23, 24). The most relevant mutations are found within the receptor-binding domain which allows the escape of NAbs induced by the vaccine (24, 25). The immune evasive potential of the Omicron variant to escape the humoral immunity generated by the vaccination schemes led to the implementation of a fourth immunization (6, 23). Our data reveal a higher immune response for the original Wuhan strain than for the Omicron variant, but even so, there is a high response to Omicron after the fourth dose, which demonstrates the effectiveness of increasing the humoral response of the booster of the SARS-CoV-2 vaccine. However, in IMRDs patients a lower response to Omicron was observed, which may be due to the pharmacological treatments used to control rheumatic disease.

Knowing the duration of humoral immunity is crucial for the control of SARS-CoV-2 infection and, therefore, for making public health decisions regarding vaccination programs. Various studies analyze the permanence of total and NAbs against SARS-CoV-2 in healthy controls (26–29). In scientific studies carried out in IMRDs patients, such as RA, a difference in the kinetics of the humoral response has been observed, where patients required 2 doses of the vaccination to reach higher levels of antibodies (30). Other articles show that patients with RA have a high seropositivity of 97% but with significantly lower total antibody levels than healthy controls (31). This information is consistent with our data since we have also observed a high antibody titer for the original Wuhan strain and the Omicron variant. According to our data, seropositivity in rheumatic patients was 99.5% for the Wuhan variant and 99% for the Omicron variant, while in controls it was 100% for both strains. However, further analysis revealed that after 35 days from vaccine administration, IMRDs patients experienced a decrease in NAbs to SARS-CoV-2. This could be due to the initial state of the immune system of IMRDs patients, who are under Immunosuppressive treatments. Therefore, the response to vaccination is not maintained over time, as is the case with healthy controls. The rapid decline in NAbs leads to reduced protection and increased risk of infection (32), which reinforces the idea to consider booster doses (30–34).

In response to the pandemic, the Chilean Ministry of Health implemented different vaccination schemes against SARS-CoV-2. After two doses of vaccines, reinforcements were made with the BNT162b2, CoronaVac or mRNA-1273 vaccines (6, 20, 35). Recent studies carried out by health workers reveal that homologous vaccination presents better results than heterologous schemes (36). Due to the increased risk of contracting severe COVID-19 by the population using immunosuppressive treatments, such as IMDRs patients, it is necessary to analyze the efficacy of vaccination against SARS-CoV-2 in this population. Studies carried out with the CoronaVac (37) and BNT162b2 (11) vaccines show good immunogenicity for IMDRs patients. However, metadata analysis studies suggest that IMDRs patients may obtain better results if they are inoculated with a homologous vaccination scheme (38). This is consistent with our results, where a homologous vaccination system showed a higher neutralizing antibody titer against SARS-CoV-2.

The humoral response of each IMRDs was evaluated, but it has limitations due to the small sample size of some diseases included, such as PsA, AS, SV and SS. The humoral response of RA patients against the parental Wuhan strain did not show differences compared to healthy controls, but against the Omicron variant, a significant decrease in the NAbs titers was observed compared to healthy controls. The other diseases included did not show differences in humoral response. The high humoral response achieved in these diseases demonstrates the effectiveness of the 4th dose of vaccine against SARS-CoV-2. The decreased response against the Omicron variant in RA patients may be secondary to immunosuppressive treatment. In a study of two-dose regimen of the BNT162b2 vaccine in patients with PsA, axial spondylarthritis, SLE and Large vessel vasculitis, the seropositive rate was above 90%. In patients with RA, the seropositive rate was 82.1%, whereas the lowest seropositive rate (<40%) was observed in patients with ANCA-associated vasculitis and idiopathic inflammatory myositis (11) Our study provides information on the impact of pharmacological treatments on the immunogenicity induced by vaccination. GC are commonly used in combination with other drugs, making it difficult to assess their effect in monotherapy. A reduction in immunity from SARS-CoV-2 vaccines has been reported in patients receiving GC therapies (11, 39). Likewise, our data reveal that the use of GC in combination with other therapies decreases the titer of NAbs against SARS-CoV-2.

The impact of MTX treatment on the immunogenic response to vaccination has been analyzed in various scientific papers, showing an adverse effect on the humoral and cellular immune response to mRNA vaccines (11, 12). Due to this, the possibility of suspending MTX two weeks before vaccination to favour the immunogenicity of the vaccine against SARS-CoV-2 has been discussed (40, 41). However, other authors point out that this suspension does not improve the response to the vaccine or could cause flares of the disease in patients who are not stable (42, 43). Our data indicates that the combined use of methotrexate with other drugs reduces the humoral immune response, but the analysis of the individual impact of the drug does not show statistical differences, although a median similar to the combined treatment is observed. Our data also reveal that other DMARDs, such as LEF and mycophenolate, are associated with decreased immunogenic response to vaccination, as has been observed in other studies (11, 44). HCQ, in combination, also decreased the humoral response in this study, but HCQ is frequently combined with MTX, so this could be an effect of MTX.

In addition to the limitations mentioned above, we can indicate the sample size of the control group, which was smaller than the group of rheumatological patients. Besides the sample size of the control group, it can be mentioned that the age of the control group was significantly lower. This was due to the vaccination process that occurred in Chile and how certain groups of the population were prioritized for the fourth dose, which meant that the vaccination times of the rheumatological group coincided with the health personnel, who on average are younger than the patients. It is important to take this bias into consideration when interpreting the results; in any case, the necessary statistical analyzes were carried out.

Biological treatments used to control rheumatic diseases and whose objective is to inhibit cytokines do not seem to interfere with the production of antibodies induced by vaccination (11). Drugs such as golimumab, etanercept, and adalimumab, which are TNF inhibitors, and secukinumab, an interleukin 17 inhibitor, are used by the patients in our study, and no significant decrease in the production of antibodies in response to vaccination against SARS-CoV-2 was observed.

In this study, treatment with rituximab and abatacept demonstrated a significant decrease in the humoral response against the Omicron variant. Following what has been reported in other studies, patients treated with rituximab and abatacept present a decrease in the immunogenic response to vaccination (11, 45–48). The lapse of time between the administration of rituximab and vaccination is a relevant factor in the immunogenic response of IMRDs patients, showing an improvement in the serological response when there is a longer interval between both administrations (11, 47).

## Conclusions

Our study provides information on the humoral response to SARS-CoV-2 vaccines in patients with IMRDs. Factors such as the different vaccination schedules, humoral response times, diagnosis, and different pharmacological treatments of IMRDs patients were considered. In IMRDs patients, the humoral response variation in the SARS-CoV-2 vaccine depends on doses and type of vaccine administered, the humoral response times and the treatment that these patients are receiving. In light of our results, it is highly relevant to carry out studies on the response to vaccination in immunosuppressed populations, since their response to vaccination differs from the general population.

## Data availability statement

The raw data supporting the conclusions of this article will be made available by the authors, without undue reservation.

## Ethics statement

The studies involving humans were approved by Proyecto: No 161-2021 Archivo acta: N° 109. UNIVERSIDAD DE CHILE - FACULTAD DE MEDICINA COMITÉ DE ÉTICA DE INVESTIGACIÓN EN SERES HUMANOS. The studies were conducted in accordance with the local legislation and institutional requirements. The participants provided their written informed consent to participate in this study. Written informed consent was obtained from the individual(s) for the publication of any potentially identifiable images or data included in this article.

## Author contributions

MG: Conceptualization, Investigation, Writing – original draft, Writing – review & editing, Data curation, Funding acquisition, Methodology, Project administration, Supervision. MC: Writing – original draft, Writing – review & editing, Investigation, Methodology. YG: Formal analysis, Investigation, Software, Supervision, Validation, Writing – original draft, Writing – review & editing, Data curation. CF: Methodology, Writing – review & editing. JS: Investigation, Writing – review & editing. LA: Data curation, Investigation, Writing – review & editing. EN: Investigation, Writing – review & editing. AG: Formal analysis, Investigation, Writing – review & editing. ER: Investigation, Writing – review & editing. AK: Resources, Writing – review & editing. RS: Investigation, Resources, Writing – review & editing. FV: Conceptualization, Funding acquisition, Investigation, Methodology, Resources, Writing – review & editing.
