# Supplementary material for: 4th booster-dose SARS-CoV-2 heterologous and homologous vaccination in rheumatological patients
Source: Front Immunol. 2024 Jul 26;15:1427501. doi: 10.3389/fimmu.2024.1427501 (PMC11309998; doi:10.3389/fimmu.2024.1427501)
Supplement: Supplementary file 1 [file DataSheet_1.docx]

Supplementary figure


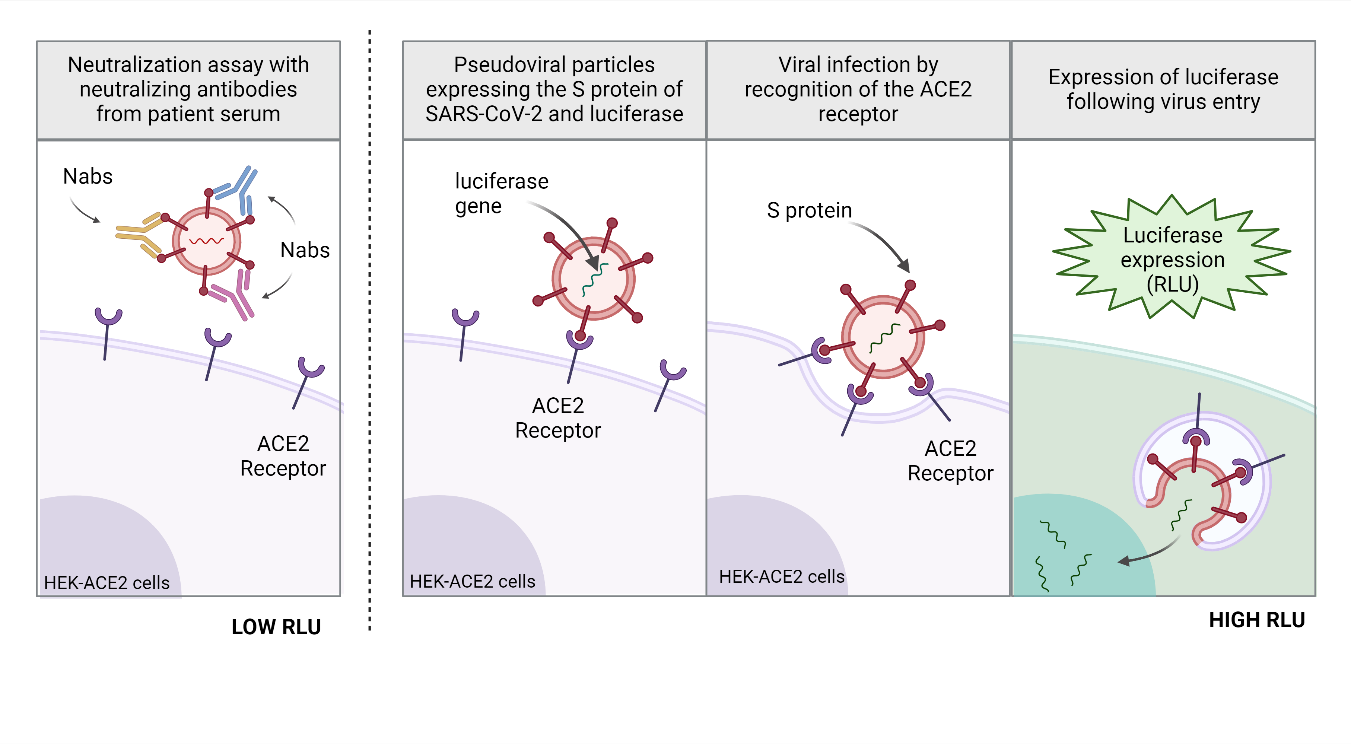


**Figure 1.** Neutralization assay. An infection assay is performed in the presence of serial dilutions of patient samples serum (1:40, 1:120, 1:360, 1:080, 1:3240, 1:9720, 1:29160, and 1:87480). As the sample dilution increases, the concentration of neutralizing antibodies decreases, thereby increasing infection and also increasing Relative Light Units (RLU). Negative control, no infection in HEK293 Wt cells, low RLU. Positive control, 100% infection in HEK-ACE2 cells, high RLU
